# Molecular Basis of Color Variation in Taiwanese Loach Revealed by Early Developmental Transcriptome Analysis

**DOI:** 10.3390/ani16121849

**Published:** 2026-06-15

**Authors:** Benhe Ma, Yan Hu, Aijun Ma, Tao Hu, Ruiyu Deng, Zhihui Huang, Haihua Wang

**Affiliations:** 1 Jiangxi Fisheries Research Institute, Nanchang 330039, China; mabenhe@126.com (B.M.);; 2 Nanchang Key Laboratory of Special Aquactic Breeding and Healthy Aquaculture, Nanchang 330039, China; 3 National Key Laboratory of Mariculture Biobreeding and Sustainable Production, Qingdao 266071, China; 4 Yellow Sea Fisheries Research Institute, Chinese Academy of Fishery Sciences, Qingdao 266071, China

**Keywords:** Taiwanese loach, red mutant, early development, body coloration, transcriptome, pigmentation genes, aquaculture industry, thyroid hormone

## Abstract

Background: The Taiwanese loach (*Paramisgurnus dabryanus* ssp. Taiwan, Dabry de Thiersant, 1872.) is an economically important aquaculture species in East Asia, and its body color directly affects its ornamental and market value. Our research group recently discovered a golden-red mutant, named “Gan Hong No. 1” (MR), within a wild-type (WT) population. During embryogenesis, MR individuals exhibit almost no melanophore deposition, and after hatching, xanthophores and erythrophores appear sequentially, suggesting that the body color variation likely originates from alterations in the gene regulatory network during early development. Objective: To systematically compare the transcriptomes of WT and MR-Taiwanese loach during early development, to identify the key regulatory pathways underlying red body color formation from a temporal perspective, to test whether the classical melanin synthesis pathway is impaired, and to provide a theoretical basis for selective breeding of body color traits. Methods: High-throughput transcriptome sequencing was performed on eight early developmental stages (0, 5, 10, 15, 20, 23, 28 and 43 h post-fertilization) of both loach types. Differential expression analysis, time-series trend analysis, and Kyoto encyclopedia of genes and genomes (KEGG) pathway enrichment were used to systematically characterize gene expression dynamics. Transcriptomic data validation was performed using real-time PCR. Results: In MR, the core transcription factor *mitfa* was significantly downregulated, whereas the expression of melanin synthesis genes such as *kita* and *dct* showed no significant difference, indicating that the impairment of melanogenesis is caused by mitfa downregulation. Trend analysis and pathway enrichment revealed that in MR embryos, pathways related to oxidative stress, unsaturated fatty acid biosynthesis, C-type lectin receptor signaling, p53 signaling, and apoptosis were significantly activated, while the thyroid hormone synthesis pathway was markedly upregulated. In WT, these pathways showed the opposite trend. qRT-PCR results were consistent with the transcriptome data. Conclusions: This study demonstrates that downregulation of *mitfa* serves as the initial trigger for red body color variation in the Taiwanese loach. This mutation impedes melanin synthesis and concurrently activates a coordinated regulatory network involving oxidative stress, immune inflammation, and thyroid hormone signaling. Accumulation of unsaturated fatty acids alleviates oxidative damage and supports carotenoid deposition, while immune signals eliminate aberrant melanocytes and promote compensatory generation of red and yellow chromatophores. The upregulated thyroid hormone further fine-tunes pigment cell differentiation. For the first time in a cobitid species, this study elucidates the *mitfa*-mediated, multi-pathway synergistic molecular mechanism driving the transition from melanin-based to carotenoid/pteridine-based red coloration in fish, thereby providing a theoretical reference for molecular breeding of body color in aquaculture.

## Simple Summary

The Taiwanese loach is an economically important fish in East Asia, valued both as food and as an ornamental species. Body color greatly affects its market price, and a newly discovered red mutant is particularly desirable. However, the genetic reasons why this mutant develops a red color instead of the typical dark wild-type color remain unclear, especially during early life. In this study, we compared gene activity between wild-type and red mutant loach embryos across eight developmental stages, from fertilization to one day after hatching. We found that in the red mutant, a key gene responsible for black pigment production is turned down, which impairs the formation of black pigment cells. At the same time, other groups of genes become more active—those involved in cell stress, inflammation, fatty acid metabolism, and thyroid hormone signaling. Together, these changes redirect pigment production toward red and yellow pigments (carotenoids and pteridines) instead of black melanin. Our results reveal a new, non-classical mechanism of color formation in fish and provide potential genetic markers that can help breeders selectively develop red-colored loach strains for the ornamental fish industry.

## 1. Introduction

The Taiwanese loach (*Paramisgurnus dabryanus* ssp. Taiwan, Dabry de Thiersant, 1872) is regarded as an important food aquaculture species in East Asia due to its high nutritional and economic value, as it is rich in protein, fat, minerals, and various vitamins. In addition to its value as a food source, wild-type (WT) Taiwanese loach is increasingly traded worldwide as an ornamental fish, and color variants such as the red mutant (MR) hold significant market value [1,2]. Body coloration is not only a key ecological trait but also significantly affects the ornamental and market value of aquatic products. Our research team recently discovered a golden-red mutant (red mutant Taiwanese loach, MR-Taiwanese loach) within an artificially cultured population of the wild-type (WT-Taiwanese loach). Compared with WT individuals, the MR-Taiwanese loach exhibits a distinct pigmentation pattern: few pigment cells are visible before hatching, and after hatching, xanthophores and erythrophores appear sequentially without evident melanophore deposition [3,4]. This striking difference suggests that the body color variation in MR-Taiwanese loach likely arises from alterations in gene regulatory networks during critical stages of early development [4].

The body coloration of fish is primarily determined by the number, quality, and spatial distribution of pigment cells (chromatophores) located beneath the dermis [5]. These chromatophores are mainly categorized into light-absorbing types (melanophores), as well as erythrophores, xanthophores, cyanophores, reflecting leucophores, and iridophores. The pigment composition and the resulting color of each chromatophore type are summarized in Table 1 [6,7]. Our previous comparative transcriptome analysis of skin tissues from wild-type Taiwanese loach and red mutant Taiwanese loach demonstrated that the tyrosinase metabolic pathway was not significantly enriched among the top 20 pathways. Moreover, *tyr* expression showed no significant difference between the two loach strains (wild-type Taiwanese loach and red mutant Taiwanese loach), whereas oca2 expression exhibited significant variation [4]. These findings indicated that body pigmentation may be modulated by non-canonical melanin biosynthesis pathways or alternative molecular regulatory mechanisms. However, the temporal expression dynamics of pigmentation-related genes during early embryogenesis remain largely uncharacterized, particularly with respect to differences between WT-Taiwanese loach and MR-Taiwanese loach and their contribution to body color variation.

In recent years, transcriptome sequencing has been widely applied in developmental biology, providing a powerful approach for elucidating dynamic gene expression profiles and regulatory signaling cascades [8,9,10,11,12]. For example, Zhou [13] identified 18 h, 24 h, and 42 h post-fertilization as critical stages for cardiovascular morphogenesis in zebrafish via transcriptome analysis. Ma [14] conducted transcriptomic profiling across seven developmental stages in channel catfish, identifying genes associated with head formation, oocyte development, intestinal epithelial growth, brain development, immune regulation, ion transport, and cell proliferation. Collectively, these studies underscore the utility of transcriptomics in elucidating the molecular mechanisms responsible for early development in fish.

In this study, we performed comparative transcriptome profiling of WT-Taiwanese loach and MR-Taiwanese loach across eight early developmental stages, using high-throughput sequencing. For the first time, we systematically analyzed the expression trends of differentially expressed genes from a temporal perspective to identify key regulatory windows and pathways controlling body color formation. Our objectives were to elucidate the molecular mechanisms underlying the color variation in MR-Taiwanese loach, to test whether the classical melanin synthesis pathway is impaired, and to provide a theoretical basis for selective breeding of pigmentation traits in loach.

## 2. Materials and Methods

### 2.1. Sampling

**Figure 1:**
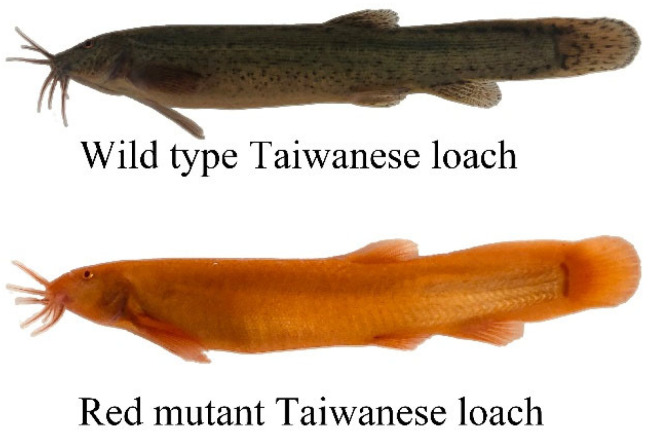
Wild-type Taiwanese loach (*P. dabryanus* ssp. Taiwan) and red mutant Taiwanese loach (red mutant strain “Gan Hong No. 1”).

Wild-type Taiwanese loach (*P. dabryanus* ssp. Taiwan, hereafter referred to as WT) and its red mutant strain “Gan Hong No. 1” (hereafter referred to as MR) were collected from Jiangxi Fisheries Research Institute (Figure 1). Healthy broodfish with intact body surface, normal growth and mature gonads were selected for artificial insemination.

The water temperature was maintained at 26 °C. Fertilized eggs were collected immediately after fertilization (0 h) as the initial samples. Sampling was subsequently conducted at 5 h intervals combined with key developmental stages, generating a total of eight sampling time points: 0 h (Stage 1), 5 h (Stage 2), 10 h (Stage 3), 15 h (Stage 4), 20 h (Stage 5), 23 h (hatching initiation, Stage 6), 28 h (complete hatching, Stage 7), and 43 h (1 day post-hatching, Stage 8). Samples of WT at each stage were coded TT1–TT8, and samples of MR were coded HT1–HT8 correspondingly.

Three biological replicates were prepared for each strain at every time point. At each sampling, no less than 300 mg of embryos or larvae was aspirated with sterile pipettes and immediately transferred into RNase-free cryotubes containing RNA stabilization reagent. All samples were incubated overnight at 4 °C and then stored long-term at −80 °C.

All animal treatments in this study were approved by the Ethics Committee (ID: YSFR1-2024019).

### 2.2. Total RNA Extraction and Library Construction

Sterile medical scissors and forceps after autoclaving were used to take WT and MR samples at different developmental stages out of the RNA stabilization reagent for total RNA isolation. Total RNA was extracted from all samples following the protocols supplied with the Tiangen Total RNA Extraction Kit (Tiangen Biotech, Beijing, China). The integrity of extracted RNA was assessed by agarose gel electrophoresis. Double-stranded cDNA was subsequently synthesized using the PrimeScript™ RT Master Mix (Takara, Beijing, China).

After end repair and adapter ligation, standard sequencing libraries were constructed. Library quality was validated via fragment size selection, quantification and quality control. Transcriptome sequencing was performed by Novogene Co., Ltd., Beijing, China, using Illumina‌.

### 2.3. Transcriptome Alignment and Differential Expression Analysis

Clean reads after quality control were aligned to the reference genome of *Paramisgurnus dabryanus* (GenBank accession number: GCA_030506205.2) using HISAT2 (V2.2.1). The featureCounts program (V2.0.1) within the Subread package (V2.0.1) was applied to count uniquely mapped reads covering the full coding region of each gene (from start codon to stop codon), and FPKM values of all genes were calculated subsequently [15].

Pearson correlation coefficients among samples were calculated based on FPKM values to assess intra-group and inter-group correlations, and heatmaps were generated using R packages (V4.5.2). Principal component analysis (PCA) was performed on gene expression data of all samples with SIMCA software (V14.1) to identify the major sources of variation. Box plots were also constructed via R packages (V4.5.2) to visualize the distribution of gene expression levels in each sample.

Differentially expressed genes (DEGs) were identified using the DESeq2 package (V1.44.0). Genes were defined as significantly differentially expressed when meeting the criteria of ∣log_10_(fold change) ∣ ≥ 1 and adjusted *p* value ≤ 0.05 [4].

### 2.4. Trend Analysis of Differentially Expressed Genes

To explore the dynamics of gene expression during early development, we integrated all differentially expressed genes (DEGs) obtained from pairwise comparisons across eight developmental stages in both wild-type (WT) and red mutant (MR) loach to construct comprehensive DEG datasets for each genotype. Genes in the *Paramisgurnus dabryanus* genome were subjected to local Basic Local Alignment Search Tool (BLAST: V2.17.0) alignment against the Pigment Biology and Core Genetic Diseases Database (https://www.ifpcs.org/colorgenes, accessed on 4 March 2024), and pigmentation-related DEGs were screened accordingly. The R package Venn Diagram (V 0.2.10‌) was used to generate Venn diagrams for visualizing the overlap of DEGs between WT and MR.

Using the Short Time-series Expression Miner (STEM) framework via the OmicShare Bioinformatics Cloud Platform (https://www.omicshare.com/, accessed on 16 January 2025), temporal clustering of gene expression was performed for WT and MR at four key developmental stages (Stage 2: 5 h post-fertilization [hpf]; Stage 4: 15 hpf; Stage 6: initial hatching; Stage 8: 1 day post-hatching) to identify gene modules with similar temporal expression patterns. Meanwhile, the temporal expression patterns of the screened pigmentation-related DEGs were specifically examined across the same four developmental stages.

### 2.5. Kyoto Encyclopedia of Genes and Genomes (KEGG) Enrichment and Gene Ontology (GO) Analysis

KEGG pathway enrichment analysis and GO analysis were performed on the differentially expressed genes (DEGs) contained in the significantly enriched modules that shared similar expression trends. The hypergeometric test (used to assess whether the enrichment of a specific gene set in functionally annotated pathways was statistically significant) was applied to identify biological pathways and categories that were significantly enriched relative to the genome-wide background [16]. For KEGG enrichment, the top 30 most significantly enriched pathways ranked by *p*-value were selected for visualization, aiming to clarify the pathways involved in the metabolic and signaling networks of *P. dabryanus*. GO analysis was performed to determine the biological functional categories of these DEGs.

### 2.6. Validation of Transcriptome Data by qPCR

Total RNA samples identical to those used for transcriptome sequencing were applied for quantitative real-time PCR (qPCR) validation. Eight pigmentation-related differentially expressed genes (DEGs), including *mitfa*, *foxd3*, *oca2*, *dct*, *kita*, *slc45a2*, *pmela*, and *creb3l2*, were randomly selected for verification (Table 2).

The corresponding gene sequences were retrieved from whole-genome resequencing data, which have been deposited into the National Genomics Data Center (NGDC) under the accession number CNP0003810 (http://db.cngb.org/cnsa/project/CNP0003810_fbf8c1fc/reviewlink/, accessed on 26 January 2026, accession number CNP0003810; data not yet publicly released). Quantitative PCR primers were designed with Primer 6.0 and synthesized by Beijing Yueda Biotechnology Co., Ltd (Beijing, China). The elongation factor 1 alpha (EF1α) was used as the reference gene, and the relative gene expression levels were calculated using the 2^−ΔΔCt^ method [17].

## 3. Results

### 3.1. Sequencing Data Quality Control and Quantitative Analysis

**Figure 2:**
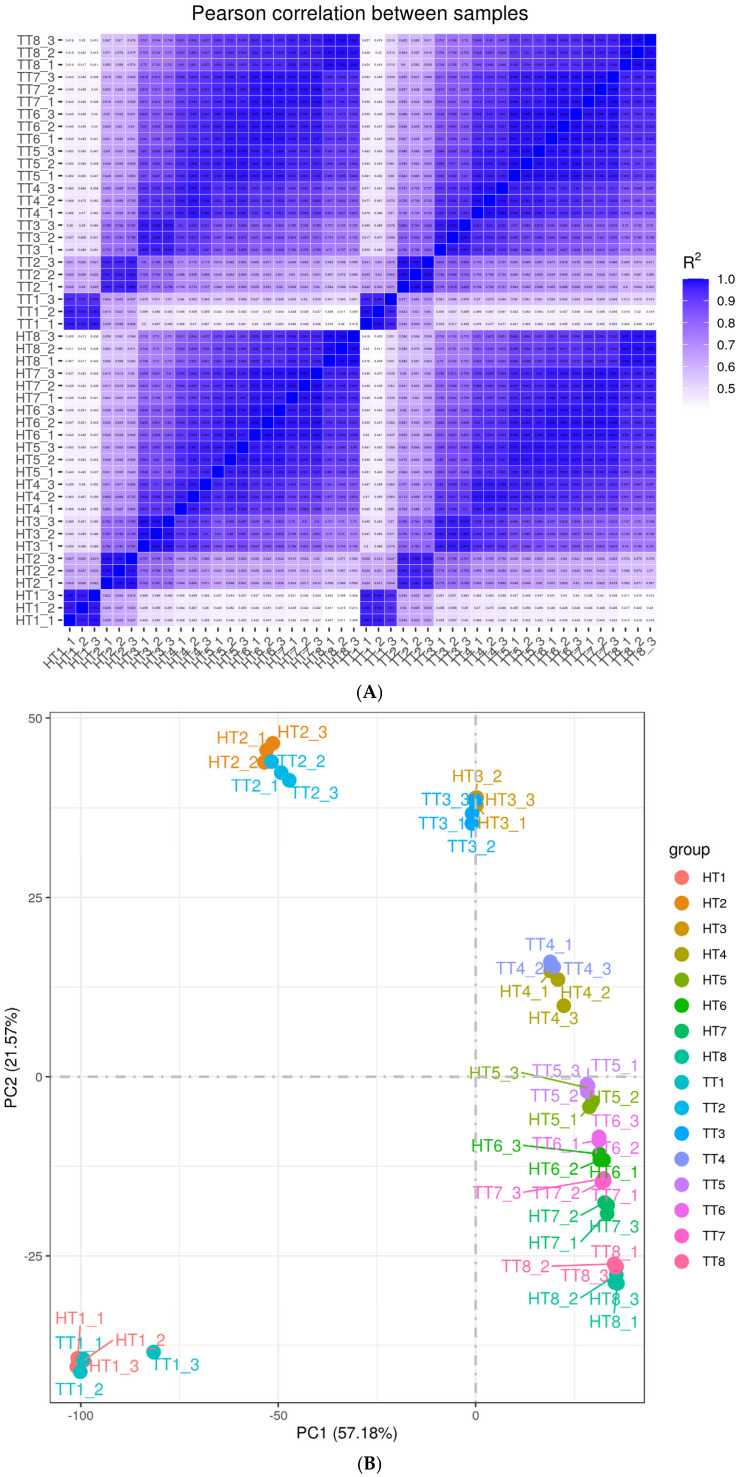
Quantitative analysis between WT-Taiwanese loach and MR-Taiwanese loach at different stages: 0 h (TT1/HT1), 5 h (TT2/HT2), 10 h (TT3/HT3), 15 h (TT4/HT4), 20 h (TT5/HT5), hatching initiation (TT6/HT6), complete hatching (TT7/HT7), and 1 day post-hatching (TT8/HT8). (**A**) Correlation heatmap; (**B**) PCA, PC1: inter-group differences; PC2: intra-group differences.

**Figure A1:**
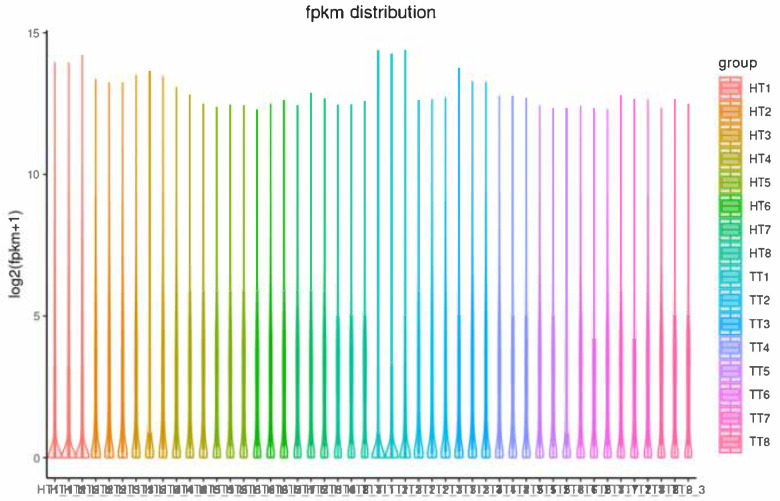
Violin plot of co-expression distribution of genes.

Statistical analysis of the transcriptome sequencing and assembly data for embryos and larvae at eight distinct developmental stages of WT-Taiwanese loach and MR-Taiwanese loach generated the following key quality control metrics. The percentages of high-quality filtered clean reads were 96.46% and 95.55%, respectively; the mean Q20 and Q30 quality scores were 97.99% and 94.38% for WT-Taiwanese loach, and 98.02% and 94.50% for MR-Taiwanese loach; and the mean GC content values were 46.28% and 45.80%, respectively. For each sample, alignment with the reference genome exceeded 85.95%. The percentage of reads uniquely mapped to the reference genome was above 80.15%, while the percentage of reads mapped to multiple genomic locations was below 6.93%. These results allowed us to proceed reliably with subsequent analyses of gene expression levels and differential expression. Gene expression levels were then quantified individually for each sample (Figure 2A). The correlation heatmap revealed that all R^2^ values between biological replicates within each species exceeded 0.95. PCA indicated good intra-group reproducibility, with samples from the same developmental stage clustering closely together (Figure 2B). In addition, violin plots were applied to visualize the distribution of gene expression at each sampling time point. The results showed that the gene expression distribution among three biological replicates in both groups was highly consistent across all time points (Appendix A, Figure A1), which confirmed good intra-group reproducibility of the experiment. Collectively, these results confirmed exceptionally high consistency among technical replicates, validating the high reliability of the data and providing a solid foundation for subsequent differential expression analysis.

### 3.2. Identification of Differentially Expressed Genes

**Figure 3:**
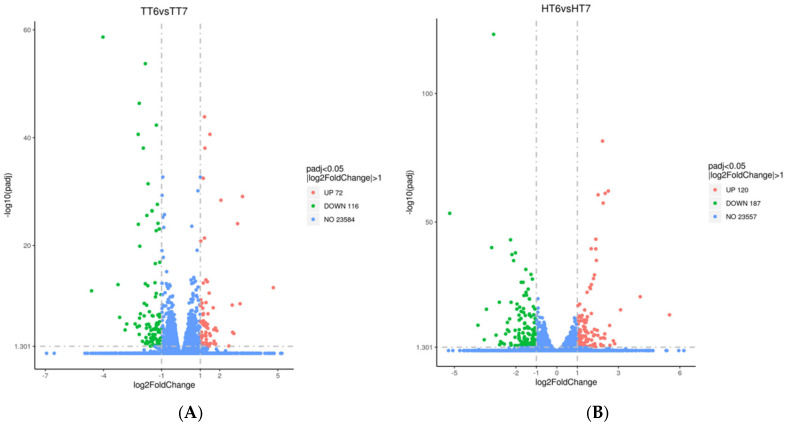
Volcano map of differentially expressed genes. (**A**) WT-Taiwanese loach; (**B**) MR-Taiwanese loach.

Pairwise comparisons of consecutive developmental stages within each strain were performed to assess transcriptional dynamics (Figure 3). We then focused on differential expression between WT- and MR-Taiwanese loach at the same developmental stage. As shown in Appendix B, the number of DEGs between the two strains at each of the eight time points was relatively low, indicating that the two strains do not exhibit major temporal shifts in their developmental progression. These inter-strain DEGs were used for subsequent trend analysis and KEGG enrichment.

### 3.3. Trend Analysis

**Figure 4:**
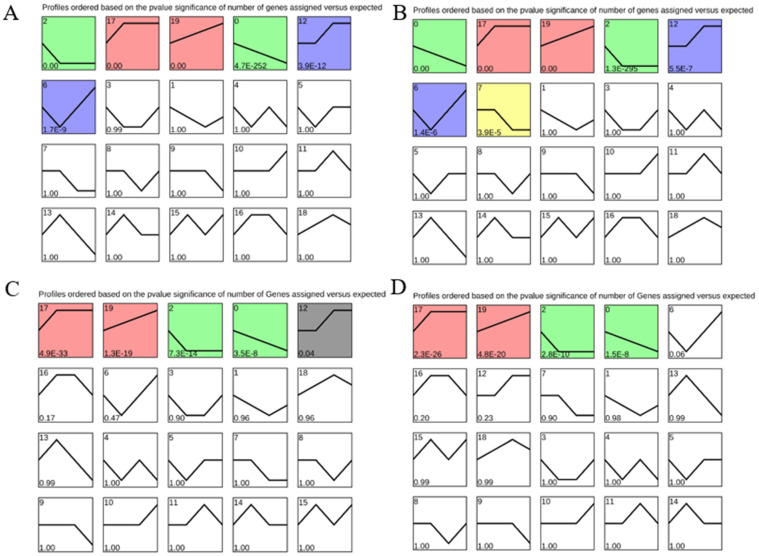
Trend analysis: (**A**) trend analysis of DEGs in WT-Taiwanese loach (HT6 vs. HT7); (**B**) trend analysis of DEGs in MR-Taiwanese loach (HT6 vs. HT7); (**C**) trend analysis of DEGs related to body color in WT-Taiwanese loach (HT6 vs. HT7); (**D**) trend analysis of DEGs related to body color in MR-Taiwanese loach (HT6 vs. HT7).

After integration and deduplication, 18,293 and 18,439 non-redundant DEGs were identified in wild-type (WT) and red mutant (MR) Taiwanese loach, respectively. All genes were clustered into 20 expression modules based on their expression trends across the four key developmental stages (Figure 4A,B). Six modules were significantly enriched in WT (Modules 0, 2, 6, 12, 17, 19), and seven significantly enriched modules were detected in MR. Modules 0, 2, 6, 12, 17 and 19 were shared by both strains, while Module 7 was uniquely present in MR.

Homology search identified 433 and 392 body color-related DEGs in WT and MR, respectively. The expression trends of these genes are presented in Figure 4C,D. In WT, Modules 17, 19 and 12 were upregulated, whereas Modules 0 and 2 were downregulated. In MR, the upregulated modules were Modules 17 and 19, and the downregulated modules were Modules 0 and 2. Venn diagrams were used to illustrate the overlap of upregulated and downregulated genes between the two strains. In total, 111 body color-related genes were co-upregulated and 57 genes were co-downregulated in both genotypes (Appendix C).

### 3.4. KEGG Enrichment Analysis and GO Analysis

**Figure 5:**
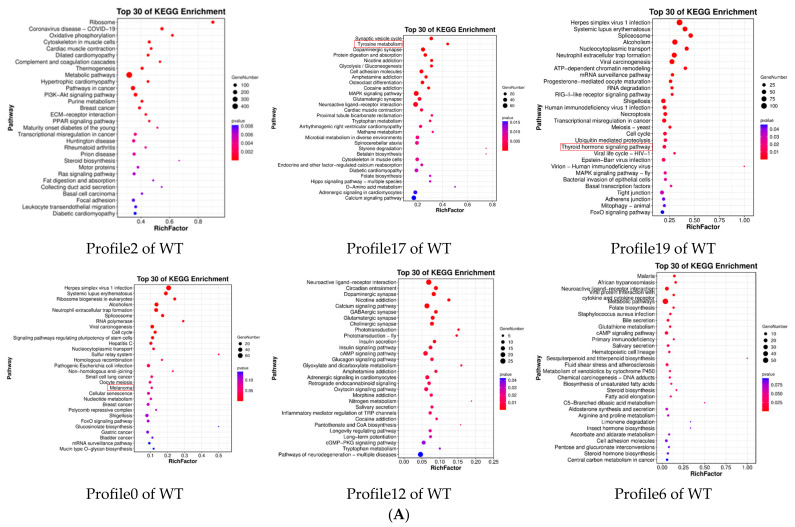

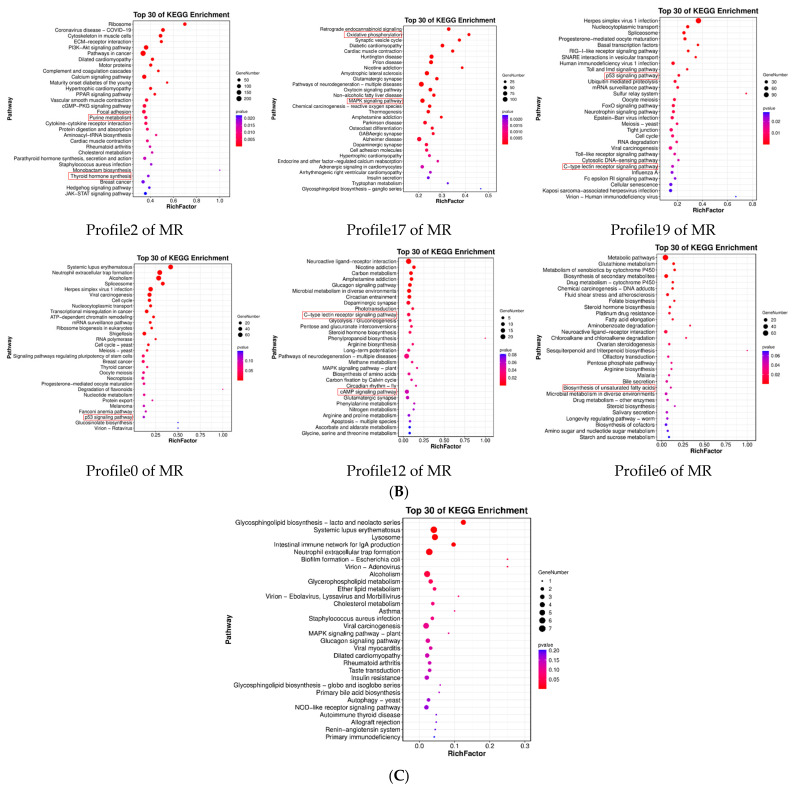
KEGG enrichment bubble plot of differentially expressed genes in modules with similar expression trends between WT-Taiwanese loach and MR-Taiwanese loach, showing only the top 30 pathways. (**A**) WT-Taiwanese loach, (**B**) MR-Taiwanese loach, (**C**) KEGG enrichment plot of the unique trend module 7 of MR-Taiwanese loach.

**Figure A4:**
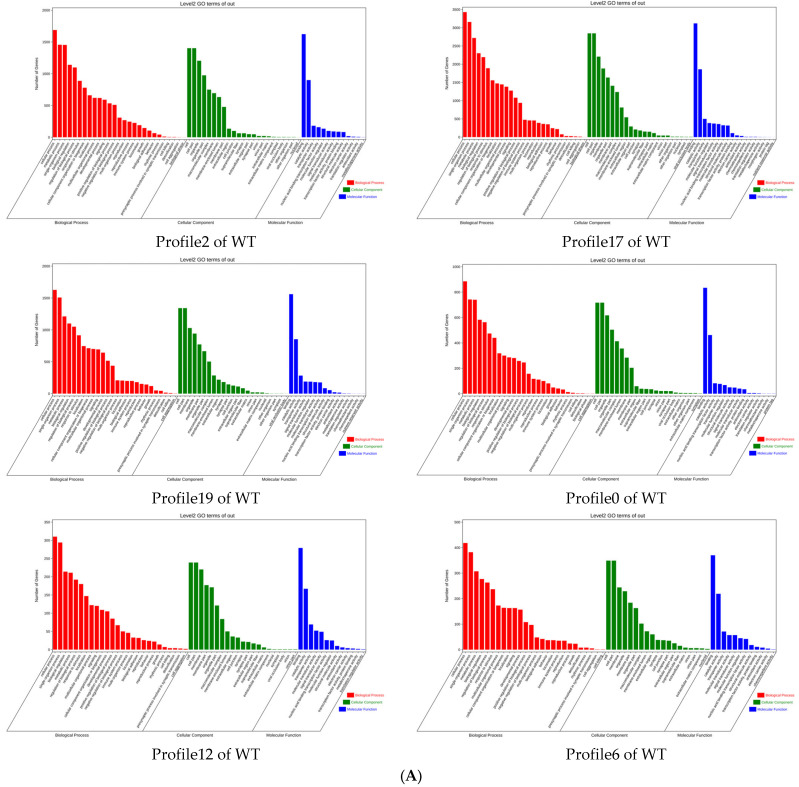

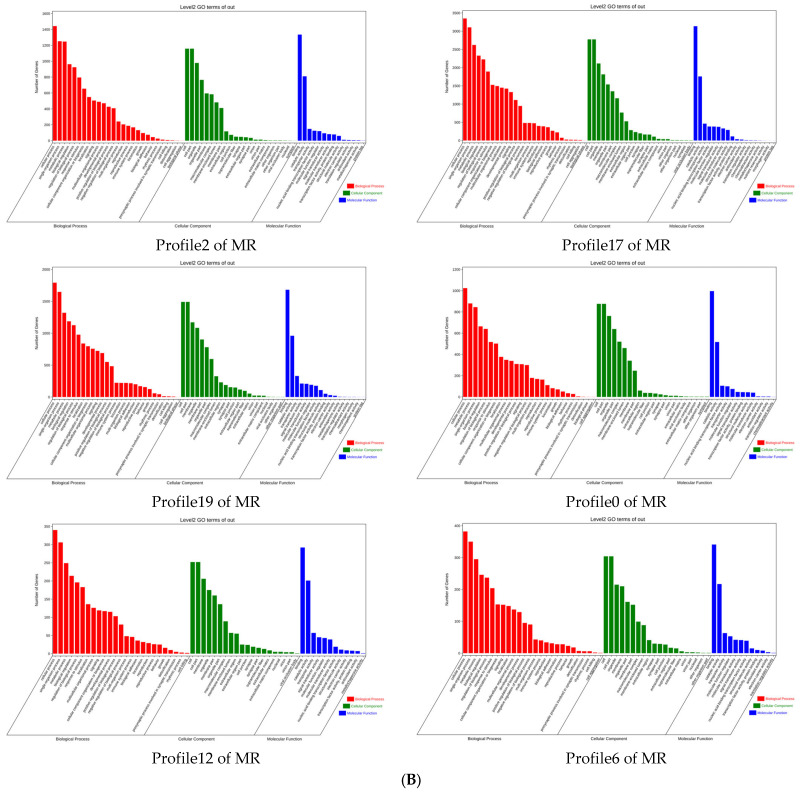
GO analysis of genes in modules with similar expression trends between wild-type Taiwanese loach and red mutant Taiwanese loach. (**A**): WT-Taiwanese loach, (**B**): MR-Taiwanese loach.

KEGG pathway enrichment analysis and GO analysis were performed on the differentially expressed genes (DEGs) within the significantly enriched modules (Figure 5 and Figure A4). The results demonstrated obvious differences in pathway enrichment preference between the two strains. In wild-type (WT) Taiwan loach, DEGs were predominantly enriched in melanogenesis-related pathways. By contrast, DEGs of the red mutant (MR) were significantly enriched in oxidative stress-associated pathways, including oxidative phosphorylation, mitogen-activated protein kinase (MAPK) signaling pathway and purine metabolism.

In addition, DEGs from MR were also enriched in the biosynthesis of unsaturated fatty acids, C-type lectin receptor signaling pathway, p53 signaling pathway and apoptosis pathway, all of which are functionally related to immune regulation. Notably, the thyroid hormone synthesis pathway was significantly upregulated in MR, while it was markedly downregulated in WT.

### 3.5. Expression Analysis of Pigmentation-Related Genes Across Developmental Stages

**Figure 6:**
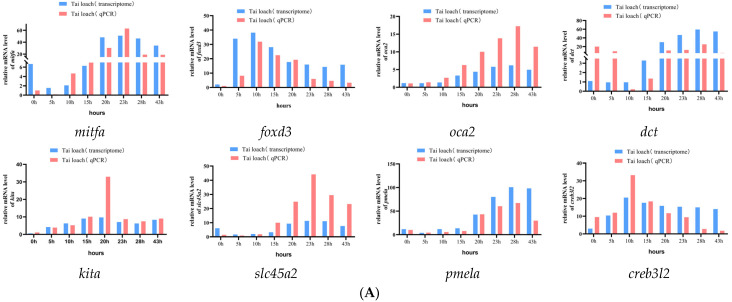

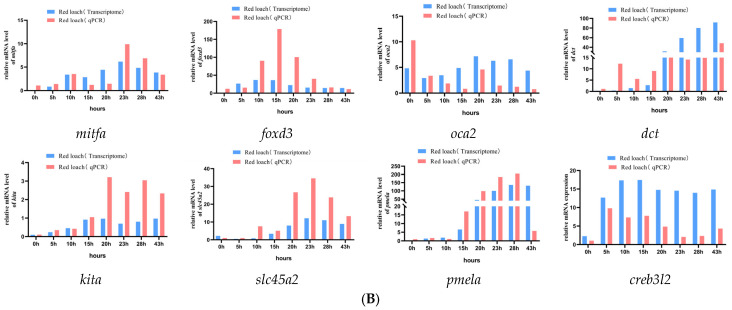
qPCR verification of DEGs between WT-Taiwanese loach and MR-Taiwanese loach. (**A**) qPCR verification of DEGs of WT-Taiwanese loach; (**B**) qPCR verification of DEGs of MR-Taiwanese loach.

To validate the RNA-seq results, eight pigmentation-related DEGs (*mitfa*, *foxd3*, *oca2*, *dct*, *kita*, *slc45a2*, *pmela* and *creb3l2*) were randomly selected for qRT-PCR analysis across the developmental stages of WT-Taiwanese loach and MR-Taiwanese loach. The relative expression levels measured by qRT-PCR were mostly consistent with the expression trends observed in the RNA-seq data, confirming the reliability and accuracy of the transcriptome results (Figure 6). Moreover, it was found that during the second half of embryonic development, the *mitfa* gene was highly expressed in WT-Taiwanese loach but exhibited low expression in the red mutant, whereas no significant differences were observed in the expression of other melanin-related genes.

## 4. Discussion

In the present study, we performed comparative transcriptome analysis across eight early developmental stages of wild-type (WT) and red mutant (MR) Taiwanese loach. The results revealed that multiple pathways related to oxidative stress (oxidative phosphorylation and purine metabolism), inflammatory response (MAPK signaling pathway and C-type lectin receptor signaling pathway), lipid metabolism (biosynthesis of unsaturated fatty acids), as well as thyroid hormone synthesis were significantly altered in MR embryos when compared with WT.

### 4.1. Defects in Melanogenesis Trigger Activation of Oxidative Stress Pathways

Multiple enriched pathways identified in the red mutant (MR) collectively indicated the activation of oxidative stress. Studies on other species have demonstrated that impaired melanogenesis disrupts intracellular redox homeostasis, which subsequently induces oxidative stress [18,19]. The biosynthesis of unsaturated fatty acids leads to the accumulation of polyunsaturated fatty acids (PUFAs) such as arachidonic acid. These compounds can partially alleviate the adverse damage caused by oxidative stress resulting from defective melanogenesis [20]. Furthermore, elevated synthesis of PUFAs facilitates the absorption and deposition of carotenoids, since these pigments primarily exist in the form of unsaturated esters in fish [21]. Consistent with previous skin transcriptome data, genes involved in the PUFA biosynthesis pathway were also upregulated in the red mutant [4]. A study by Song, L. et al. on body color variation in the crimson snapper (*Lutjanus erythropterus*) further confirmed that upregulation of unsaturated fatty acids is highly correlated with astaxanthin deposition in the skin [22]. Collectively, the oxidative stress signature and upregulation of unsaturated fatty acids observed in MR embryos provide a stress-related explanation for the shift from melanin-based pigmentation to carotenoid/pteridine-based pigmentation.

### 4.2. Immune Regulation Induces Apoptosis of Melanogenesis-Defective Cells

The significant enrichment of the C-type lectin receptor signaling pathway, p53 signaling pathway and apoptosis pathway in the red mutant (MR) indicated the activation of immune and inflammatory responses. This process is likely to remodel the differentiation and distribution of chromatophores through immune signals. When melanocytes in MR suffer from defective melanogenesis, the organism activates the relevant pathways. Cytokines released by the immune network interact with melanocytes and eventually induce cell apoptosis. Meanwhile, a greater number of xanthophores and erythrophores are generated to compensate for the loss of melanocytes, which contributes to the red body color of MR. This inference is supported by a series of previous studies on zebrafish [23,24,25].

### 4.3. Thyroid Hormone Synthesis: A Key Regulatory Switch

This study identified significant differential expression of the thyroid hormone synthesis pathway between two strains of the Taiwanese loach: the pathway was markedly upregulated in the red mutant (MR) and markedly downregulated in the wild-type (WT). This suggests that the thyroid hormone may play a central regulatory role in body coloration establishment in MR. In zebrafish (*Danio rerio*), the thyroid hormone (TH) exerts a bidirectional regulatory effect on two types of neural crest-derived pigment cells: it suppresses melanophore population expansion while promoting the proliferation of xanthophores/erythrophores pigment cells [26]. Another study demonstrated that clownfish (*Amphiprioninae*) can modulate iridophore development by altering internal TH levels, thereby adjusting the timing and number of white bars on the body surface to adapt to environmental changes [27]. Using the spotted scat (*Scatophagus argus*) as a model, Liao et al. combined exogenous thyroid hormone analogs and thyroid inhibitors with transcriptome analysis and confirmed that TH simultaneously regulates three pigment cell types—melanophores, iridophores, and erythrophores [28]. These cross-species findings collectively underscore the critical function of the thyroid hormone in fish pigmentation. The differential expression of the TH pathway revealed in this study extends the thyroid hormone–pigmentation regulatory paradigm from model fish such as zebrafish and medaka (*Oryzias latipes*) to the species-rich loach family (Cobitidae), providing new experimental evidence that thyroid hormone serves as a universal endocrine regulatory hub for body color diversification in teleosts.

### 4.4. Differential Expression of mitfa Impairs Melanogenesis in Red Mutant Loach

Microphthalmia-associated transcription factor acts as a core regulator of melanocyte development and the melanogenesis pathway. It can directly activate the expression of genes involved in melanin synthesis and transport, including *tyr* (tyrosinase), *tyrp1*, *dct* (dopachrome tautomerase) and *slc45a2*. Both RNA-seq and qPCR results revealed that *mitfa* was highly expressed in wild-type Taiwanese loach, whereas its expression was markedly reduced in the red mutant. In contrast, no obvious expression differences were detected for other melanogenesis-related genes such as *foxd3*, *oca2*, *dct* and *kita*. Collectively, these findings demonstrated that the suppression of melanogenesis in the red mutant was caused by the downregulation of *mitfa*. This conclusion is consistent with previous studies on zebrafish and koi carp [29,30].

In summary, the body color variation in MR-Taiwanese loach is primarily triggered by the downregulation of *mitfa*. The resulting defect in melanin synthesis subsequently activates synergistic pathways involving oxidative stress, immune/inflammatory responses, and thyroid hormone synthesis, collectively driving the transition of chromatophore composition from melanin-dominated to carotenoid/pteridine-dominated pigmentation.

## 5. Conclusions

This study revealed, through comparative transcriptome analysis, that the core mechanism underlying body color formation in the red mutant Taiwanese loach is the significant downregulation of the *mitfa* gene, which directly impairs the melanin synthesis pathway. This defect subsequently triggers oxidative stress and immune-inflammatory responses: on one hand, the upregulation of the unsaturated fatty acid pathway alleviates the oxidative damage caused by impaired melanogenesis and provides a material basis for the accumulation and deposition of astaxanthin; on the other hand, it induces apoptosis of melanocytes and drives compensatory proliferation of erythrophores and xanthophores. Additionally, the markedly upregulated thyroid hormone synthesis pathway acts as a key endocrine regulatory hub, synergistically inhibiting melanocyte development and promoting the proliferation and differentiation of xanthophores/erythrophores. In summary, the synergistic interaction among oxidative stress, immune regulation, and thyroid hormone signaling ultimately shifts the body color pattern of Taiwanese loach from the traditional melanin-dominant blackish coloration to a carotenoid/pteridine-dominant red coloration.

## Figures and Tables

**Table 1:** Pigments, pigment granules, and colors of six chromatophore types.

Pigment Cell Type	Pigment	Pigment Granules	Color
Melanophore	Melanin	Melanin granules	Black–brown
Erythrophore	Carotenoids and pteridines	Carotenoid vesicles, pteridine granules	Red–orange
Xanthophore	Carotenoids and pteridines	Carotenoid vesicles, pteridine granules	Yellow–orange
Cyanophore	Cyanophore pigment	Unknown	Blue
Leucophore	Leucophore pigment (urates)	Uric acid	White, silvery (reflective)

**Table 2:** qPCR primers for differentially expressed body color genes.

Number	Gene Name	Primer	TM/°C
1	*mitfa*	F: CCGCAGAAGAACAGGAACAA R: TAGGGGAGCCAGATGAGAGC	58.9
2	*foxd3*	F: AGAGCCCGCAAAAAAAGTT R: GGGGTCGAGGGTCCAGTAG	57.95
3	*oca2*	F: TAGACAACATACCCTTCACTGCGAC R: CAGAGGCTCCAATCAGAGTCCC	54.37
4	*dct*	F: GTAAATTTGGCTGGACGGG R: GGCAATGACATAATCTGGGTG	57.85
5	*kita*	F: ATGAACGAGAAAGGGACCAG R: TAACCACCCGCAGAGACAGA	57.6
6	*slc45a2*	F: CTCTACACCATCCCATACAACC R: CACCACTACAATGACACTGCC	56.05
7	*pmela*	F: TCACTCCAAGCCCTGACTG R: AAAAAATGCGATGGTTCCC	56.8
8	*creb3l2*	F: AATGGGATGGGCAGGTTGAT R: ACTTTTGCGGTCGTGTTTGA	60.15
9	*EF1α*	F: ACAGCAAGAACGACCCACC R: AAAGCGACCAAGAGGAGGAT	58.34

**Figure A2:**
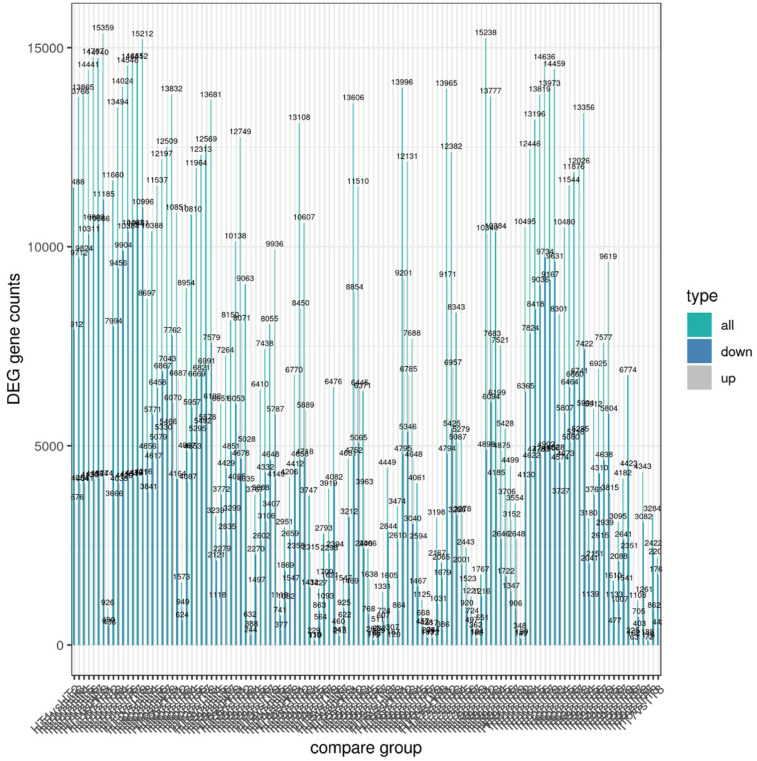
Statistical bar chart of DEGs numbers in WT-Taiwanese loach and MR-Taiwanese loach across eight developmental stages.

**Figure A3:**
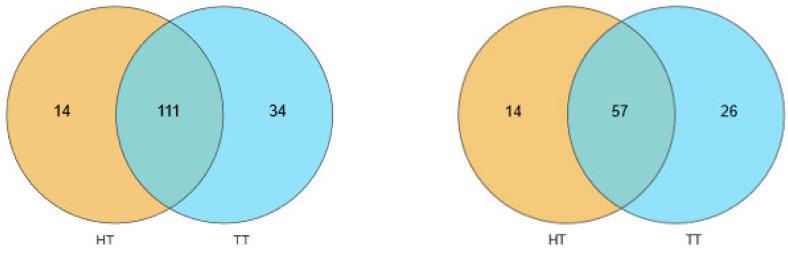
Venn diagrams of up- and downregulated genes related to body color in WT-Taiwanese loach and MR-Taiwanese loach.

## Data Availability

Data will be made available on request.

## Appendix E

**Table A1:** Abbreviations list.

Number	Abbreviation	Full Name
1	Bcl-2	B-cell lymphoma 2
2	BLAST	Basic Local Alignment Search Tool
3	cDNA	Complementary DNA
4	creb3l2	cAMP-responsive element-binding protein 3-like protein 2
5	DEGs	Differentially expressed genes
6	DESeq2	Differential Expression analysis for Sequence count data (version 2)
7	dph	Days post-hatching
8	dct	Dopachrome tautomerase
9	EF1α	Elongation factor 1 alpha
10	FPKM	Fragments Per Kilobase of transcript per Million Fragments mapped
11	foxd3	Forkhead box protein D3
12	GO	Gene Ontology
13	HISAT2	Hierarchical Indexing for Spliced Alignment of Transcripts 2
14	hpf	Hours post-fertilization
15	HT1–HT8	MR-Taiwanese loach samples stages 1–8
16	KEGG	Kyoto Encyclopedia of Genes and Genomes
17	kita	KIT proto-oncogene receptor tyrosine kinase
18	MAPK	Mitogen-activated protein kinase
19	MEK	MAPK/ERK Kinase
20	MITF	Microphthalmia-associated transcription factor
21	mitfa	Microphthalmia-associated transcription factor a
22	MR	Red mutant Taiwanese loach
23	NF-κB	Nuclear factor kappa B
24	NRAS	Neuroblastoma RAS viral oncogene homolog
25	oca2	Oculocutaneous albinism 2 protein
26	p53	Tumor protein p53
27	PCA	Principal component analysis
28	PCR	Polymerase chain reaction
29	PUFAs	Polyunsaturated fatty acids
30	pmela	Premelanosome protein
31	qPCR	Quantitative polymerase chain reaction
32	qRT-PCR	Quantitative real-time polymerase chain reaction
33	RNA-seq	RNA sequencing
34	ROS	Reactive oxygen species
35	STEM	Short Time-series Expression Miner
36	slc45a2	Solute carrier family 45 member 2
37	TGF-β1	Transforming growth factor β1
38	TT1–TT8	WT-Taiwanese loach samples stages 1–8
39	WT	Wild-type Taiwanese loach

Note: Sorted alphabetically by initial letter.
